# Characterization of the Bat proteins in the oxidative stress response of *Leptospira biflexa*

**DOI:** 10.1186/1471-2180-12-290

**Published:** 2012-12-13

**Authors:** Philip E Stewart, James A Carroll, David W Dorward, Hunter H Stone, Amit Sarkar, Mathieu Picardeau, Patricia A Rosa

**Affiliations:** 1 Laboratory of Zoonotic Pathogens, Rocky Mountain Laboratories, National Institute of Allergy and Infectious Diseases, National Institutes of Health, 903 South 4th St, Hamilton, Montana, 59840, US; 2 Laboratory of Persistent Viral Diseases, Rocky Mountain Laboratories, National Institute of Allergy and Infectious Diseases, National Institutes of Health, 903 South 4th St, Hamilton, Montana, 59840, US; 3 Research Technologies Branch, Rocky Mountain Laboratories, National Institute of Allergy and Infectious Diseases, National Institutes of Health, 903 South 4th St, Hamilton, Montana, 59840, US; 4 Institut Pasteur, Unité Biologie des Spirochètes, 25 rue du Dr. Roux, Paris, 75015, France

## Abstract

**Background:**

Leptospires lack many of the homologs for oxidative defense present in other bacteria, but do encode homologs of the *Bacteriodes* aerotolerance (Bat) proteins, which have been proposed to fulfill this function. Bat homologs have been identified in all families of the phylum *Spirochaetes*, yet a specific function for these proteins has not been experimentally demonstrated.

**Results:**

We investigated the contribution of the Bat proteins in the model organism *Leptospira biflexa* for their potential contributions to growth rate, morphology and protection against oxidative challenges. A genetically engineered mutant strain in which all *bat* ORFs were deleted did not exhibit altered growth rate or morphology, relative to the wild-type strain. Nor could we demonstrate a protective role for the Bat proteins in coping with various oxidative stresses. Further, pre-exposing *L. biflexa* to sublethal levels of reactive oxygen species did not appear to induce a general oxidative stress response, in contrast to what has been shown in other bacterial species. Differential proteomic analysis of the wild-type and mutant strains detected changes in the abundance of a single protein only – HtpG, which is encoded by the gene immediately downstream of the *bat* loci.

**Conclusion:**

The data presented here do not support a protective role for the *Leptospira* Bat proteins in directly coping with oxidative stress as previously proposed. *L. biflexa* is relatively sensitive to reactive oxygen species such as superoxide and H_2_O_2_, suggesting that this spirochete lacks a strong, protective defense against oxidative damage despite being a strict aerobe.

## Background

Molecular oxygen freely diffuses across bacterial membranes and can give rise to damaging reactive oxygen species (ROS) such as superoxide radicals (O_2_^−^), hydrogen peroxide (H_2_O_2_), and hydroxyl radicals (OH·). These highly reactive molecules lead to a variety of harmful effects within the bacterial cell, including inactivation of Fe-S-containing proteins and damage to DNA and to lipids, in some bacteria. For aerobic microorganisms the presence of these toxic species is by nature unavoidable and they have therefore evolved a variety of protective enzymes to preemptively detoxify ROS.

The enteric bacteria have been intensively studied for their response to ROS (recently reviewed by [1]). In contrast, leptospires lack a number of the enzymes used by enteric bacteria to combat oxidative damage [2] and are also more susceptible to H_2_O_2_-mediated killing than other microorganisms [3]. Nascimento and colleagues speculated that the Bat proteins of *L. interrogans* might partially compensate for the shortage of oxidative stress proteins by providing an additional line of defense against oxidative damage [2].

The Bat proteins were first identified by Tang and co-workers in a transposon mutagenesis screen of the anaerobe *Bacteroides fragilis*[4]. The transposon inserted into the fourth of five contiguous open reading frames and resulted in reduced levels of aerotolerance and pathogenicity. The genes were designated *bat*, for Bacteriodes aerotolerance genes, and were shown to comprise an operon. The mutant phenotype could be partially complemented by the addition of reducing agents and the Bat proteins were proposed to directly reduce oxidatively-damaged proteins in the periplasm or, alternatively, to help create a reduced environment in the periplasm by exporting reducing power equivalents. Interestingly, anaerobic growth did not restore the growth rate to that of wild-type and the addition of reducing agents also increased growth of the wild-type strain, although not as dramatically as it did for the mutant.

Recently, two *bat* homologs in *Francisella tularensis* were inactivated and the *bat* mutants were shown to have a reduced ability to replicate in macrophage cells and were also attenuated for virulence in a mouse model [5]. The specific function of the Bat proteins, however, was not determined in *F. tularensis*. Genome sequences have identified homologs in a wide variety of other prokaryotes, including all families that comprise the phylum *Spirochaetes* (*Brachyspiraceae*, *Leptospiraceae*, and *Spirochaetaceae*). Although conserved in all branches of the *Spirochaetes*, the number and combination of *bat* homologs vary by species. However, the function of the Bat proteins in spirochetes or in any other species has not been elucidated.

Although pathogenic leptospires also contain *bat* homologs and are more resistant to peroxide exposure than the saprophyte *L. biflexa*[3,6], the pathogenic spp. are notoriously recalcitrant to targeted allelic exchange. Since *L. biflexa* is more amenable to genetic manipulation than pathogenic species, it serves as a model organism for genetic studies in leptospires. Therefore, we used *L. biflexa* to investigate the function of the Bat proteins and to better understand the response of leptospires to oxidative stress. Here, we report the engineered deletion of the three contiguous *L. biflexa bat* genes and characterization of the mutant phenotype and oxidative stress response.

## Results

### The *bat* genes are distributed throughout the *Spirochaetes* and encode conserved protein motifs

Homologs of the *bat* genes are present in each family of the *Spirochaetes* (Additional file 1: Figure S1), although not in all species. In contrast to the 5 genes present in *B. fragilis*, *L. biflexa* contains 3 *bat* genes and the pathogenic leptospires contain 4 [2,7-9]. However, the *batB* and *batC* genes are fused in *L. biflexa*, which does not appear to be the case for the pathogenic species, and explains the discrepancy in gene number. Fusions of *bat* coding regions also appear to have occurred in *Borrelia burgdorferi* and *Spirochaeta thermophila* (Additional file 1: Figure S1) and were also reported for *F. tularensis* type A strain Schu S4 [5].

**Figure 1:**
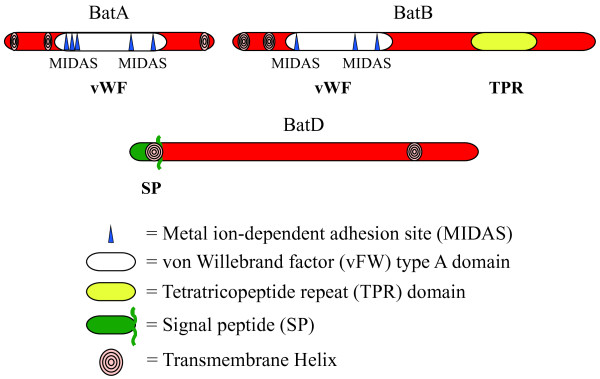
**Amino acid motifs in the Bat proteins of *L. biflexa*.** The vWF and TPR domains are conserved among Bat homologs and have been proposed to facilitate formation of a large Bat protein complex [4]. The vWF domains identified in Bat proteins contain metal ion-dependent adhesion sites (MIDAS) shown to bind metal ions [10] and the domain overall is thought to mediate protein-protein interactions [11]. The TPR domain of BatB consists of a repeated amino acid motif previously shown to form a tertiary scaffold structure for multiprotein complex formation (reviewed in [12]). These domains, along with the presence of multiple transmembrane helices and a signal sequence identified in BatD, suggest that the Bat proteins form a complex associated with either the inner or outer membrane of *L. biflexa*.

Analysis of BatA and BatB sequences identified motifs predicted to mediate protein-protein interactions, (Figure 1). The presence of these motifs suggest that Bat proteins may interact with each other to form a large protein complex. All three proteins are predicted to contain multiple trans-membrane helices, also predicted for the *B. fragilis* homologs, and BatD possesses a predicted signal sequence for export, suggesting that these proteins may associate with either the inner or outer membrane of *L. biflexa*.

### Deletion of *bat* genes

**Figure 2:**
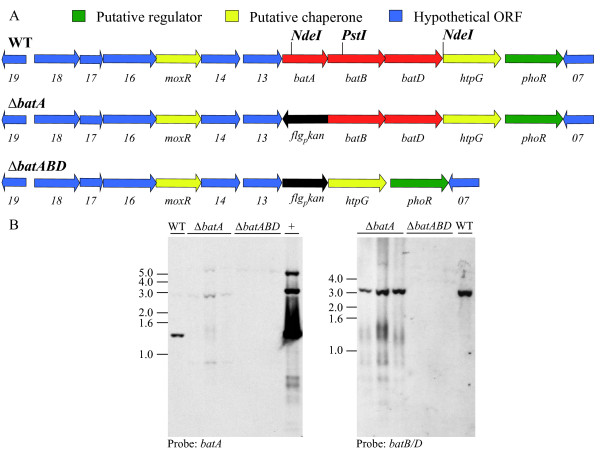
**Gene organization in wild-type and mutant strains of *L. biflexa*.** (**A**) Genetic organization of *bat* genes and flanking genes on chromosome II of *L. biflexa* (not drawn to scale). The corresponding deleted regions in mutant strains are depicted with the respective *bat* genes replaced by the kanamycin-resistance cassette [13]. (**B**) Southern blot analysis of *L. biflexa* strains confirms the absence of the respective *bat* genes in mutant strains. Genomic DNA for the Southern blot was double-digested with restriction endonucleases NdeI and PstI. Three independently isolated transformants from each mutant were compared to wild-type and hybridized with either a labeled *batA* fragment or with a labeled fragment spanning *batB* to *batD*. The weak signal observed at ~3 kb in the *batA* mutant strains hybridized with the *batA* probe is likely due to cross-hybridization with *batB*. +, purified plasmid DNA from *E. coli* with a cloned region of *L. biflexa* DNA containing *batABD*.

The *L. biflexa bat* genes are located within a contiguous stretch of 11 genes on chromosome II that are transcriptionally oriented in the same direction (Figure 2A). Two different mutations were engineered using allelic replacement with the kanamycin-resistance cassette to delete either *batA* alone or *batABD* together; flanking genes were left intact. Three mutant clones from each transformation were shown to have lost the corresponding *bat* loci by Southern blot analysis of genomic DNA (Figure 2B). PCR analysis also confirmed the presence of the antibiotic-resistance gene (*kan*) and flanking genes, but *bat* loci were absent, as expected (data not shown). A single transformant of each type was randomly chosen for further characterization.

### Transcript analyses indicate independent promoters in the *bat* gene cluster

**Figure 3:**
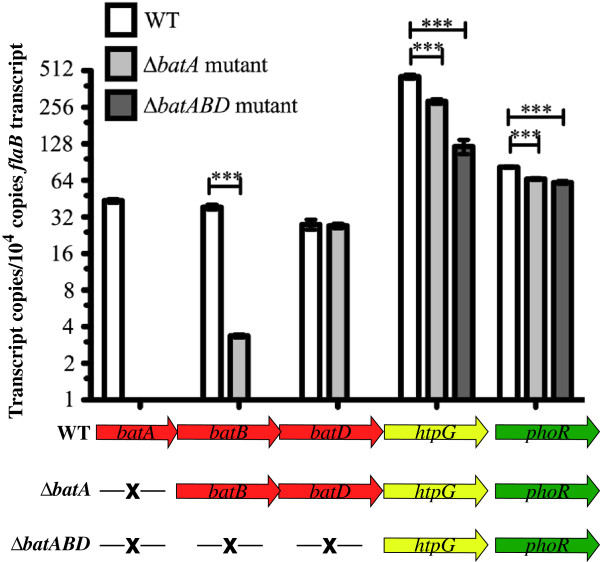
**Quantitative RT-PCR analysis of the *bat* locus and downstream genes.** Gene targets are shown below the corresponding section of the bar-graph using specific primer-probe sets for each gene (Table 1). Transcript from each gene was normalized to 10^4^ copies of *flaB* transcript from the respective strain. –X–, indicates deletion of the corresponding gene indicated above. Values represent the mean of triplicate reactions ± the standard error. Unpaired T test with Welch’s correction was used to determine significant differences between two groups (e.g. *batB* transcript levels between WT and Δ*batA* mutant strains). For statistical analysis of more than 2 groups (such as comparisons of gene transcripts between WT, Δ*batA* mutant and Δ*batABD* mutant strains), one-way analysis of variance (ANOVA) with the Bonferroni’s post test was applied. P values < 0.0001 are denoted by ***.

Transcript levels of *bat* genes and other ORFs were assessed by qRT-PCR with RNA from wild-type (WT), Δ*batA* and Δ*batABD* strains cultured in vitro (Figure 3). Transcript from the *bat* genes is present in the WT strain but undetectable in the *ΔbatABD* mutant, as expected. In the Δ*batA* mutant strain, only the *batA* transcript is undetectable, but transcripts from the downstream ORFs, including *batB* and *batD*, were detected. Although the arrangement of the 11 genes suggest they may be co-transcribed in an operon, the deletion of the *bat* genes does not eliminate transcript from the downstream ORFs and we hypothesize that each gene has an independent promoter. Interestingly, even ORFs immediately downstream of the deleted genes had observable levels of transcript, even though their promoter regions were most likely located in the deleted sequences. However, the levels of transcript from the downstream genes were significantly lower in the mutant strains compared to transcript levels in the WT: *htpG* transcript levels were 3.7-fold lower in the Δ*batABD* strain, and *batB* transcript levels were >12-fold lower in the Δ*batA* mutant.

### Morphology and growth rate of *bat* mutants are equivalent to wild-type

**Figure 4:**
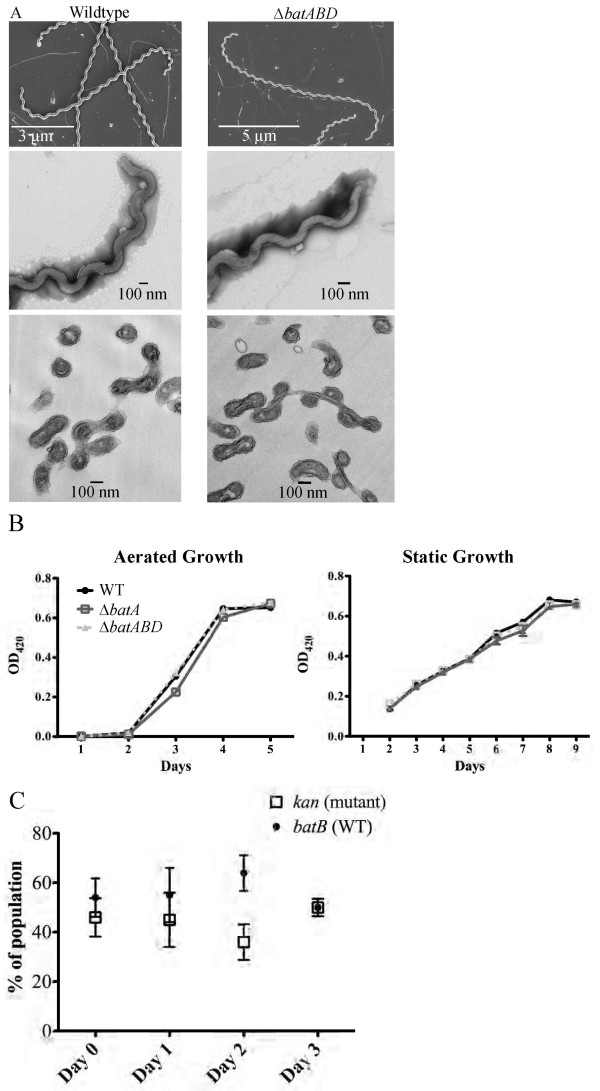
**Deletion of *bat* loci does not alter morphology or growth of *L. biflexa*.** (**A**) Electron micrographs of WT and mutant *L. biflexa* strains. No difference was observed in the morphology of the mutant strains relative to the WT (*batA* images not shown). Top panel – SEM images of *L. biflexa* strains; middle panel – TEM negative stains of spirochetes; bottom panels – TEM cross-section images of spirochetes. (**B**) Growth curves of *L. biflexa* strains grown with shaking (aerated cultures) or without shaking (static cultures). Data represent the mean ± the standard error calculated from quadruplicate cultures. (**C**) Results of co-growth of wild-type and Δ*batABD* mutant in the same culture. Aerated cultures were sampled daily to determine the percent of wild-type cells (·) and of Δ*batABD* mutant cells (□) in the population. Both strains remained at about the same percentage of the population throughout the timecourse, indicating that the Δ*batABD* mutant did not show a competitive disadvantage during in vitro cultivation. Variations over time were not statistically significant as determined by 2-way ANOVA. Data represent the mean ± the standard error calculated from triplicate cultures.

The signal sequence of BatD suggests a periplasmic or membrane-associated location for at least one member of this protein family. We therefore examined whether the absence of Bat proteins affected cellular shape or structure. *L. biflexa* morphology was assessed by scanning and transmission electron microscopy, including negative stains and freeze-substitution fixation to retain a more native state of the cells. As shown in representative images in Figure 4A, no morphological or ultrastructural differences were observed between the WT and mutant strains by any of these analyses.

Growth rates of WT, Δ*batA*, and Δ*batABD* strains were compared during in vitro cultivation in EMJH liquid medium and also for colony formation on solid EMJH medium. No significant differences in growth rate were observed when cultured in liquid medium, regardless of whether the cultures were aerated or static (Figure 4B). Colony morphology and rate of formation were similar among all strains (data not shown).

As the mutant strains did not display an obvious growth defect compared to WT, we assessed the growth dynamics of both parent and mutant when cultured together in the same medium (Figure 4C). WT and Δ*bat-ABD* strains were co-inoculated into the same cultures (performed in triplicate) and assessed daily to determine if population ratios changed over time. As shown in Figure 4C, relative proportions of each strain did not change significantly over time and this was statistically confirmed by two-way Analysis of Variance (ANOVA) with the Bonferroni post-test. Therefore, the Bat proteins do not significantly affect *L. biflexa* growth, either in pure culture or when the mutant is mixed with an equal density of WT cells.

### Deletion of *bat* genes does not alter tolerance to oxidative stress

**Figure 5:**
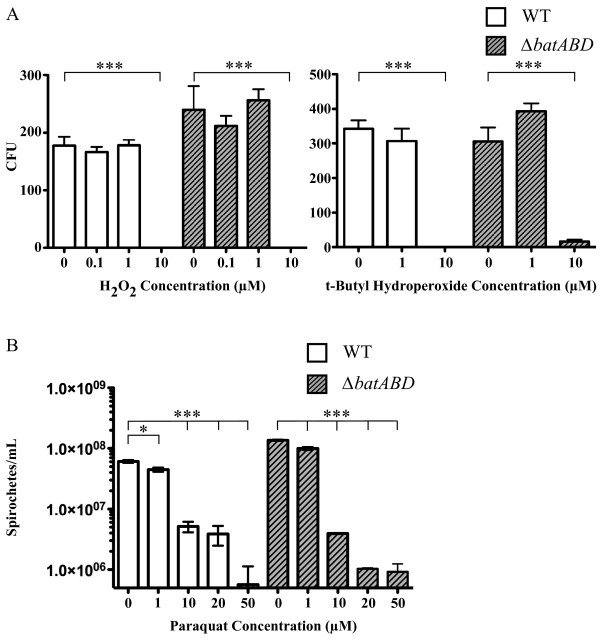
**Susceptibility of *L. biflexa* strains to ROS.** (**A**) WT or Δ*batABD* strains were exposed to varying concentrations of either H_2_O_2_ (left panel) or to the more stable organoperoxide, *tert*-Butyl hydroperoxide (right panel) and viability determined by counting CFUs after plating. (**B**) Effect of paraquat concentrations on *L. biflexa*. Twenty four hours after exposure to varying concentrations of the superoxide generator paraquat, viability was assessed by counting motile spirochetes using darkfield microscopy. One-way ANOVA was used to determine significant differences between treated and untreated samples (* denotes P value < 0.05, *** denotes P value < 0.0001). Values represent the mean ± the standard error.

Previous researchers speculated that Bat proteins might provide a mechanism for coping with oxidative stress [2,4,14]. Therefore, we compared the resistance of WT and Δ*batABD* strains to various concentrations of hydrogen peroxide and a more stable organic peroxide (*tert*-Butyl hydroperoxide), and to superoxide. We utilized the Δ*bat-ABD* mutant in this comparison as we hypothesized that it would have a similar or greater phenotype than the single gene deletion in the Δ*batA* strain. Both the WT and the Δ*batABD* strain exhibited comparable levels of susceptibi-lity to all ROS tested, with greater than 90% killing when exposed to 10 μM concentrations of H_2_O_2_, but resistant to 1 μM (Figure 5A). Similarly, when *L. biflexa* strains were exposed to paraquat, a redox-cycling compound that generates superoxide, WT and mutant strains displayed similar susceptibility to paraquat concentrations (Figure 5B).

### *L. biflexa* lacks an inducible stress response to ROS 

**Figure 6:**
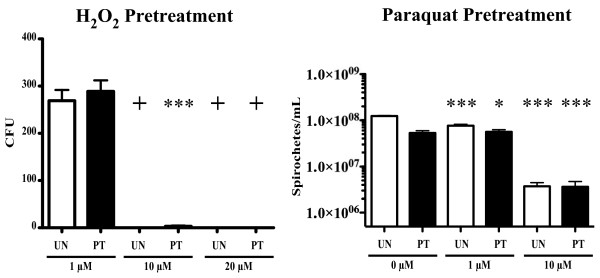
**Effect of ROS pretreatment on viability of *L. biflexa* exposed to lethal concentrations of ROS.** WT *L. biflexa* was pretreated with sub-lethal levels of H_2_O_2_ (left panel) or superoxide generated by paraquat (right panel) and compared to samples that were not pretreated. Subsequently, cultures were exposed to varying concentrations of ROS and viability assessed by either colony counts on solid medium (H_2_O_2_) or by enumerating motile spirochetes using a Petroff-Hauser counting chamber and darkfield microscopy (paraquat). UN, untreated cells; PT, pretreated cells. One-way ANOVA was used to determine significant differences between treated and the respective untreated samples (* denotes P value < 0.05, *** denotes P value < 0.0001,). Values represent the mean ± the standard error. **+** denotes that statistical analysis was not performed because the value was zero and a standard error could not be calculated.

Bacteria such as *E. coli* and *Salmonella typhimurium* exhibit an inducible response to oxidative agents [15,16]. When activated by exposure to sublethal levels of oxidizing agents, this stress response allows some bacteria to induce enzymes that allow the cell to survive otherwise lethal levels of oxidants. As the Bat proteins did not aid in resistance to oxidative stress, we next tested whether their function may relate to sensing oxidizing agents and inducing a specific stress response in *Leptospira*. Mid-to-late log phase cultures were incubated in sublethal concentrations of either H_2_O_2_ (1 μM) or paraquat (0.5 μM) to potentially induce an oxidative stress response. Cultures were then subjected to various concentrations of ROS that included normally lethal levels, further incubated, and viable bacteria enumerated (Figure 6). Surprisingly, both pretreated and untreated cells were sensitive to similar concentrations of ROS, indicating that *L. biflexa* does not exhibit an inducible response to either H_2_O_2_ or superoxide. The Δ*batABD* mutant strain was likewise treated but did not show any differences from the WT with either pretreatment (data not shown).

### Differential proteomic analysis of WT and mutant strains detected changes in HtpG levels among membrane-associated proteins

**Figure 7:**
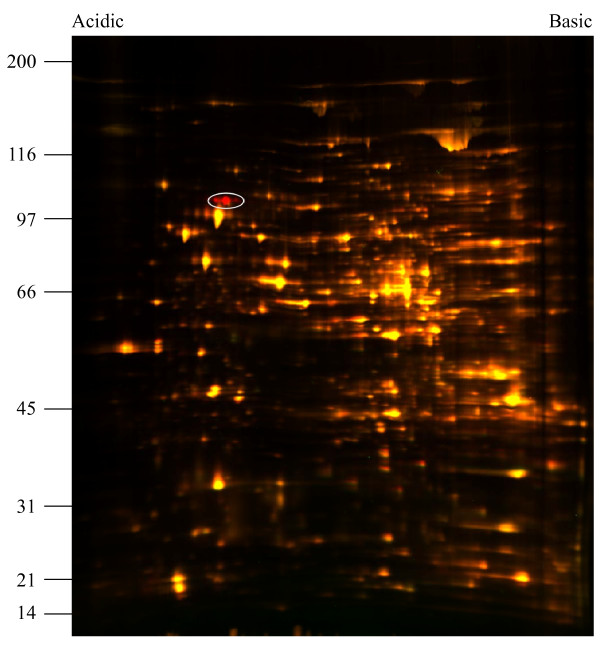
**Two-dimensional differential in-gel electrophoresis of WT and mutant membrane-associated proteins.** WT protein was labeled with Cy5 (red) and protein from the Δ*batABD* strain was labeled with Cy3 (green). Proteins present in equivalent amounts appear yellow, those present in larger amounts in the WT appear red, and proteins in higher amounts in the mutant appear green. White oval indicates a series of closely-migrating proteins that are down-regulated in the Δ*batABD* strain relative to the WT. These proteins were identified as HtpG. Relative molecular mass markers are shown to the left in kDa.

Differential in-gel electrophoresis (DIGE) of WT versus Δ*batABD* mutant protein samples was used to identify changes in protein levels between strains (Figure 7). Protein samples were separated into membrane-associated and soluble fractions. No differences were observed in the soluble fractions (data not shown) but in the membrane-associated comparison, a single protein was observed to vary between samples (Figure 7, white oval). The region encompassing the protein was excised from the gel, trypsin-digested and identified by mass spectrometry as HtpG (40 peptides detected, 61% coverage), which is encoded by the gene immediately downstream of *batD* (Figure 2A). The HtpG protein appeared as several closely migrating spots, with the main mass of protein indicated in Figure 7. Protein levels were higher in the WT compared to the Δ*batABD* strain, and differences for each spot ranged from 2.7-fold for the minor spot to greater than 4-fold higher for the main protein spot. This difference in HtpG protein levels approximately corresponds to the difference observed in transcript levels by qRT-PCR between WT and Δ*batABD* strains (Figure 3). The Bat proteins were not identified by this approach. Bat protein levels may be relatively low and the fold change between mutant and WT may not be significant enough to be detected by the conditions tested here. For example, transcript levels of *htpG* in the WT strain are more than 10-fold higher than any of the *bat* transcripts (Figure 3).

## Discussion

Bat homologs are present in all families of the *Spirochaetales* (Additional file 1: Figure S1), despite the vast evolutionary divergence noted in this order [17]. The retention of these proteins suggests they confer an evolutionary advantage to spirochetes, even though the environment and life cycle of these bacteria are incredibly diverse, ranging from free-living aerobic saprophytes (*L. biflexa*) and anaerobic thermophiles (*Spirochaeta thermophila)* to mammalian pathogens (*L. interrogans* and *B. burgdorferi*). *L. borgpetersenii*, purportedly undergoing genome reduction, retains the same number and order of *bat* genes as *L. interrogans*[7], again suggesting the Bat proteins provide an important function that prevents their elimination even in a decaying genome. One spirochete appears to be an exception to this theory – the obligate human pathogen and syphilis agent, *Treponema pallidum*, in which we were unable to identify any Bat homologs.

In addition to the wide-spread distribution of the Bat proteins in the *Spirochaetales*, MoxR and HtpG are also encoded in many spirochete genomes (data not shown). The MoxR chaperone is postulated to coordinate the metal ion into the Bat proteins MIDAS domain (Figure 1) [18]. In the sequenced *Leptospira* genomes*, moxR* and *htpG* are located in the same contiguous gene cluster as the *bats* (Figure 2A) [2,7-9]. However, Dieppedale et al. inactivated *moxR* in *F. tularensis* and their proteomic comparisons of wild-type to the *moxR* mutant did not identify changes in Bat protein levels [5]. HtpG is a homolog of the eukaryotic heat shock protein Hsp90, but its function in bacteria is unclear and it has been reported to have different roles in different prokaryotes [19-22]. The arrangement of the 11 tandem ORFs in this cluster suggest they potentially form a large operon, but qRT-PCR analyses detected transcript from the ORFs downstream of the deleted *bat* genes. The presence of transcript from the downstream ORFs, regardless of the orientation of the inserted kanamycin-resistance cassette, implies that these genes can be independently transcribed (Figure 3). These data do not rule out the possibility of an additional promoter that drives expression of all 11 genes in an operon, but do support independent promoters for the genes downstream of the deleted regions.

Somewhat surprisingly, transcript from genes immediately following the deletion site had detectable levels of transcript, although these levels were significantly lower than WT levels. Specifically, transcript of *batB* was detected in the Δ*batA* strain, even though the endogenous promoter is likely to be located in the deleted *batA* gene. However, *batB* transcript levels are over 10-fold lower in the Δ*batA* strain compared to wild-type, suggesting that the kanamycin-resistance cassette upstream of *batB* may provide a weak, fortuitous promoter sequence. A similar result was also observed for *htpG* transcript in the Δ*bat-ABD* strain; presumably, the *htpG* promoter would be located in the deleted region. The borrelial *flgB* promoter used to drive *kan* expression in the deletion of *batABD* is oriented in the same transcriptional direction as the endogenous genes (specifically, *htpG*) and read-through may account for the *htpG* transcript detected, albeit at a lower level than the endogenous promoter would produce.

The presence of a signal sequence, transmembrane helices and motifs for protein-protein interactions, also conserved in the Bat proteins of *Leptospira* (Figure 1), led Tang et al. to propose that the Bat proteins of *B. fragilis* formed a complex in the periplasm [4]. Despite their putative cellular location, growth rate and morphology of *L. biflexa* were unaffected by the loss of these proteins (Figure 4). Nor could we demonstrate a protective role for the Bat proteins in coping with oxidative stress, as initially proposed for *B. fragilis* and subsequently hypothesized for other spirochetes [2,14]. The wild-type and Δ*batABD* mutant were equally susceptible to oxidative challenge by both peroxides and the superoxide generator paraquat (Figure 5), indicating that the Bat proteins do not contribute to *L. biflexa*’s limited ability to cope with oxidative damage. However, the lack of an observable phenotype for the *bat* mutants may relate to in vitro growth where the transcript levels for these genes is quite low relative to *flaB* or *htpG* transcript levels (Figure 3). It is conceivable that *bat* expression may increase under specific in vivo conditions of which we are unaware. Various microarray studies, however, did not detect any significant changes in *bat* transcript levels in pathogenic leptospires when in vitro conditions were altered to mimic in vivo environments [23-29].

We also examined the potential contribution of the Bat proteins to sensing ROS and inducing an oxidative stress response in *L. biflexa*. Enteric bacteria such as *E. coli* and *Salmonella typhimurium* have well-characterized oxidative stress responses that can be induced by the addition of sublethal levels of peroxide [15,16] or superoxide [30-32]. However, pretreatment of exponentially growing *L. biflexa* cultures with either 1 μM H_2_O_2_ or 0.5 μM paraquat failed to confer a higher level of resistance to ROS when subsequently challenged with lethal levels (Figure 6). Therefore, it appears that *L. biflexa* does not have the same capability as enteric bacteria of inducing an oxidative stress response, at least under the conditions tested. *L. biflexa* lacks homologs for the two main regulators of the oxidative stress response in enteric bacteria (SoxR and OxyR), in support of this conclusion. However, *Leptospira* spp. do possess a PerR homolog (LEPBI_I2461 in *L. biflexa*), a negative regulator of peroxide defense first characterized in Gram positive bacteria (reviewed in [33]). Lo et al. reported a PerR transposon mutant of *L. interrogans* that resulted in an 8-fold increase in resistance to hydrogen peroxide over the wild-type [25]. However, microarray data of this mutant did not report any significant changes in *bat* transcript, suggesting that these genes may not be under the regulatory control of PerR. It is still possible that the Bat proteins are involved in sensing ROS, but the cellular response they may direct remains enigmatic.

Surprisingly, even wild-type *L. biflexa* is highly susceptible to oxidative stress compared to *B. burgdorferi* (10 μM vs. 10 mM, respectively, for t-*Butyl* hydroperoxide) [34] or *E. coli*[35]. The relative susceptibility of *L. biflexa* to oxidative damage may be due to the absence of some proteins capable of detoxifying ROS or repairing damaged proteins. For example, *L. biflexa* does not have recognizable homologs of glutathione reductase, thioredoxin 2, Ferric reductase, and others. However, *L. biflexa* does possess both superoxide dismutase (Sod) and KatG (a Hydroperoxidase I enzyme), two enzymes widely conserved among aerobic organisms for defense against ROS. Sod catalyzes the reduction of O_2_^−^ to H_2_O_2_ and O_2_. Hydrogen peroxide is itself an oxidant that freely diffuses across membranes and can give rise to the highly reactive hydroxyl radical. Therefore, aerobic cells require a mechanism for detoxifying H_2_O_2_. Catalase or peroxidase enzymes usually fulfill this cellular function and a gene encoding KatG, which can have either activity, has been identified in the *L. biflexa* genome (LEPBI_I2495). Since catalase activity has not been detected in *L. biflexa* strains but peroxidase activity has [36-40], it seems likely that KatG is a peroxidase and provides a mechanism by which *L. biflexa* detoxifies H_2_O_2_, albeit not very effectively. *L. biflexa* also possesses alkyl hydroperoxide reductase homologs (LEPBI_I3008 & LEPBI_I3009) that may also detoxify H_2_O_2_. Superoxide dismutase may play an essential role in *L. biflexa’s* defense against oxidative stress, as we were unable to inactivate the *sod* gene, either by allelic exchange or by transposon mutagenesis (data not shown).

Finally, we employed a proteomic comparison of wild-type and mutant spirochetes to identify *L. biflexa* proteins whose expression may be altered due to the loss of the Bat proteins. Two-dimensional differential gel electrophoresis of protein lysates from the wild-type and the Δ*batABD* strain identified HtpG as the sole protein in the *ΔbatABD* strain that had significantly reduced levels compared to the wild-type (Figure 7). Altered levels of HtpG were detected in the membrane-associated protein fraction, but not the soluble fraction (data not shown), although HtpG does not have any recognizable signal or lipidation sequences. However, Lo et al. also reported that HtpG associated with the membrane fraction in their analyses of temperature effects on protein levels in *L. interrogans*[24]. In our analysis, HtpG was downregulated approximately 4-fold in the Δ*batABD* mutant relative to the WT, and this decrease corresponded to the 3.8-fold decrease in *htpG* transcript levels observed by qRT-PCR (Figure 3), discussed above. Although HtpG protein is lower in the mutant, this variation did not produce a phenotype in the conditions tested here.

## Conclusions

*L. biflexa* has a relatively small repertoire of enzymes for defense against ROS, and it may depend on the activities of Sod and KatG to survive oxidative assault. During in vitro growth, *bat* transcript levels are relatively low and deletion of the *bat* loci did not detectably alter morphology, growth rate, or the ability to survive oxidative stress. Despite the proposed role for the Bat proteins in directly combating oxidative damage in spirochetes, the data presented here do not support this. Although we cannot exclude a role for the Bat proteins in sensing oxidative stress in *L. biflexa*, perhaps as a signaling complex in the periplasm, Bat function remains elusive.

## Methods

### Bacterial strains used in this study

*L. biflexa* serovar Patoc strain Patoc I (kindly provided by Dr. Dave Haake and Dr. Marija Pinne) was cultured at 30°C with shaking at 150 RPM in EMJH medium (Fisher Scientific, Pittsburgh, PA) [41,42]. Plating medium included 1.2% wt/vol Noble agar (final concentration) (Fisher Scientific) and plates were incubated at 30°C, inverted and sealed with parafilm. Kanamycin, when needed, was added to the medium at a final concentration of 20 μg/mL. *Escherichia coli* strains TOP10 (Invitrogen, Carlsbad, CA) or NEB5α (New England Biolabs, Ipswich, MA) were used for all plasmid manipulations.

### Construction of *L. biflexa* mutant strains

Transformation of *L. biflexa* followed the protocol of Louvel and Picardeau [43]. *L. biflexa* deletion mutants were constructed by allelic exchange with the kanamycin-resistance marker driven by the borrelial *flgB* promoter [13]. Proof-reading polymerases Vent (New England BioLabs) or the Expand Long Template PCR System (Roche Applied Science, Indianapolis, IN) were used for fragment amplification according to the manufacturer’s recommendations and the fidelity of amplification was confirmed by double-stranded sequencing. Primers used for plasmid construction are shown in Table 1. The region encompassing the *batABD* locus and surrounding sequences was PCR-amplified using primers Lb.htpG.F and Lb.II0014.RC, yielding a 6,113 bp fragment that was then cloned into pCR-XL-TOPO (Invitrogen). Inverse PCR was used to delete the *batABD* genes using primers batKO.F.NheI and batAKO.RC.NheI, which incorporated NheI restriction enzyme sites for self-ligation of the resulting product. NheI restriction enzyme sites were also incorporated onto the kanamycin–resistance cassette by PCR amplification using primers Pflg.NheI.F and Tkan.NheI.RC. Both the pTopoXL::ΔbatABD and the *flgB*_*P*_*-kan* cassette were digested with NheI and ligated together to create the allelic exchange vector pΔABD1-kn. A similar strategy was used to create the allelic exchange construct for *batA* (pΔbatA-kn) using primers batB.seq1.F and Lb.II0013/14.PCR1.RC to amplify a 2,565 bp fragment containing *batA*. Inverse PCR with primers batAKO.F.NheI and batAKO.RC.NheI were used to delete the coding region of *batA* and engineer the restriction enzyme sites needed to insert the kanamycin-resistance cassette. The deletions of the respective *bat* genes in the mutant strains of *L. biflexa* were confirmed by Southern blot analysis of total genomic DNA digested with the restriction enzymes NdeI and PstI, as previously described [44,45]. Primers used for probe amplification are listed in Table 1.

**Table 1:** Oligonucleotides used in this study

**Oligonucleotide**	**Sequence (5**^**′**^**– 3**^**′**^**)**	**Function**
Lb.htpG.F	GTCTACATTGAGATGGATGTGG	Amplification of *batABD* + flanking sequences
Lb.II0014.RC	CAGACCAATTACTCAAATGC	Amplification of *batABD* + flanking sequences
batB.seq1.F	CAGCGATGGACTCTAGAAAATC	Amplification of *batA* + flanking sequences
Lb.II0013/14.PCR1.RC	CTGTTGTTATCTTCGCTTCAC	Amplification of *batA* + flanking sequences
batAKO.RC.NheI	^*a*^gctagcGTTAGGTTATAAAATCCTTTTTG	Construction of allelic-exchange plasmids
batKO.F.NheI	gctagcCATATGCAAGCTGAAGAAAAAGG	Construction of Δ*batABD* allelic-exchange plasmid
batAKO.F. NheI	gctagcATGGAAACAAATACGGTTATTTAC	Construction of Δ*batA* allelic-exchange plasmid
Pflg.NheI.F	gctagcTACCCGAGCTTCAAGGAAGATT	Amplification of *kan*
Tkan.NheI.RC	gctagcGAGCTAGCGCCGTCCCGTCAA	Amplification of *kan*
Lb.batA.F	CTGGGAACTGAGTTTCTTGG	Amplification of *batA* probe
Lb.batA.RC	CTCGTCCTATCATCCTACAGG	Amplification of *batA* probe
Lb.batB.RC	CCAGAACCAATCCAATGGGC	Amplification of *batB/D* probe
batD.PCR1.RC	GAATTCGACTTCGACCGAG	Amplification of *batB/D* probe
flaB.F.qPCR	CTGCTTACAGGAGCGTTTGCT	qPCR primer
flaB.RC.qPCR	TGGTGCATGTTAGCTCCAATATG	qPCR primer
flab.Lb.Probe	^*b*^ACTCAACCCAACTGCTAGTATGTGGTT	qPCR probe
batA.F.qPCR	AGGAGCCGCATACTTACAATCC	qPCR primer
batA.RC.qPCR	GGATGTACCGGCTATCAGTTCAT	qPCR primer
batA.probe	^*b*^CTTTCAAGTGACCGTTTTGCCT	qPCR probe
batB.F.qPCR	CCTGGAACCGGGAAAGGT	qPCR primer
batB.RC.qPCR	ATCACATTGTCGCCGTAAGGT	qPCR primer
batB.probe	^*b*^CTTTGTTACTTACGATTCTAATTTGGTAG	qPCR probe
batD.F.qPCR	TGTCGCTATGGTAGAAGGATTCG	qPCR primer
batD.RC.qPCR	TGCGGACACTCCCTGTTTC	qPCR primer
batD.probe	^*b*^AAAGAAATTACTTCCTCTCTGAGTTCTTAG	qPCR probe
htpG.F.qPCR	TTTTCGGGAGCAACTGACTTC	qPCR primer
htpG.RC.qPCR	TCCTAGTCCAAAATGGCCTATGAT	qPCR primer
htpG.probe	^*b*^CCAAACAGTACCAGAACACAGAAAATAAGGCAG	qPCR probe
phoR.F.qPCR	CGTTTGATTCGCAGGGTGAT	qPCR primer
phoR.RC.qPCR	TTAGGCTCCAAGGCAGATAAAATT	qPCR primer
phoR.probe	^*b*^AAGCGGTGCAAACTGCACTCAATTTTG	qPCR probe

^*a*^Restriction enzyme sequences designated in lower case letters.

^*b*^TaqMan probes were labeled at the 5^′^-end with FAM (6-carboxyfluorescein) and at the 3^′^-end with TAMRA (6-carboxytetramethylrhodamine).

### RNA isolation and quantitative RT-PCR analysis

Total RNA was isolated from 10 mL cultures of exponentially growing *L. biflexa* cells using TRIzol reagent (Invitrogen). Cells were pelleted at 7,000 RPMs in 15 mL Falcon 2059 tubes and the pellet resuspended in 5.0 mL TRIzol. After incubation at room temperature for 2.5 min with vigorous shaking, 1 mL of chloroform was added, mixed and incubated for a further 2.5 min. The suspension was centrifuged again and the aqueous phase removed to a new Falcon tube and the RNA precipitated by addition of 5 mL isopropanol. Following a 10 minute incubation (room temperature), RNA was pelleted, washed in 75% ethanol and dissolved in 100 μL of RNase-free water. DNA was removed by treating with Turbo DNase (Ambion, INC. Austin, TX) following the manufacturer’s recommendations.

RNA was converted to cDNA using the High-Capacity cDNA Reverse Transcription kit (Applied Biosystems, Foster City, CA); reaction mixtures consisted of 1 μg RNA and were converted to cDNA per the manufacturer’s recommendations. The cDNA samples were diluted 1:20 with water and 2 μL used for subsequent quantitative PCR (qPCR) reactions. All samples were analyzed in triplicate. TaqMan Universal PCR Master Mix kit (Applied Biosystems) and PCR conditions were as previously described [46]. *L. biflexa* genomic DNA ranging from 10^6^ cells to 10 cells (in 10-fold serial dilutions) was used to generate a standard curve using the Ct values from the *flaB* primer/probe set. This standard curve was then used to interpolate the number of transcript copies from the Ct values generated from gene-specific primer/probe sets. The resulting transcript levels were then normalized to 10^4^ copies of *flaB* transcript. Negative controls lacking reverse transcriptase were included to demonstrate that all genomic DNA had been degraded and did not contribute to the signal.

### Electron microscopy, growth rate analysis and oxidative stress assays

Bacterial suspensions from cultures grown in EMJH media were prepared for scanning electron microscopy (SEM) essentially as described previously [47]. Samples were lightly sputtered with iridium and examined on a model SU-8000 scanning electron microscope operated at 2 kV (Hitachi High Technologies America, Pleasanton, CA). Images were digitized using the on-board frame card according to the manufacturer’s specifications.

For transmission electron microscopy (TEM), bacteria were prepared as described previously for imaging by microwave-assisted processing [48]. Grids were examined using a model H-7500 transmission electron microscope, operated at 80 kV (Hitachi). Digital images were captured and recorded using a model HR100 camera system (Advanced Microscopy Techniques, Danvers, MA).

Growth rate comparisons were performed in quadruplicate. Five mL cultures were inoculated at 10^5^ cells/mL from a starter culture grown to between 5 × 10^8^ to 1 × 10^9^ cells/mL, as determined by counting with Petroff-Hauser counting chambers. All cultures were incubated at 30°C; aerated cultures were shaken at 150 RPM. Cell densities were measured by optical density at 420 nm in a spectrophotometer.

Co-growth comparisons of wild-type and mutant strains were similarly tested with each strain inoculated at 10^5^ cells/mL in the same culture (for a combined concentration of 2 × 10^5^ cells/mL). Aliquots were removed daily from triplicate cultures, counted and diluted appropriately for colony formation on non-selective EMJH agar plates. PCR was performed on 24–30 colonies per plate to enumerate wild-type and mutant cells by amplifying a fragment of *batB* (wild-type) and the kanamycin-selectable marker (mutant).

Oxidative stress assays were also performed similarly. Peroxide-treated cultures were first diluted to 10^3^ cells/mL and peroxides were then added at specified concentrations and incubated for approximately 2 ½ hours, after which 100 μL samples were removed from each culture and spread on EMJH agar plates. After 4–6 days of incubation at 30°C, plates were removed and colony counts used to calculate viable cells. A similar strategy was followed for assessing whether an oxidative stress response could be induced in *L. biflexa*; quadruplicate cultures of 10^3^ cells/mL were exposed to a sublethal level of H_2_O_2_ (1μM) for 3 hrs with aeration, followed by the addition of specified concentrations of peroxide and a further incubation for 3 hours. Aliquots of 100 μL were removed and spread on EMJH agar plates to determine viable cell counts. Superoxide sensitivity was determined by diluting triplicate cultures to 5 × 10^6^ cells/mL and exposing to various concentrations of the superoxide-generating molecule paraquat (Sigma-Aldrich, St. Louis, MO) with incubation for 24 hrs. Cell viability was determined by counting motile cells using a Petroff-Hauser chamber with darkfield microscopy. To determine if *L. biflexa* produces an oxidative stress response to superoxide, triplicate cultures of 5 × 10^6^ cells/mL were pre-exposed to 0.5 μM paraquat for 2.5 hrs followed by addition of specific concentrations of paraquat. Cultures were further incubated for 24 hrs and cell viability assessed as described above.

### Two-dimensional differential in-gel electrophoresis (2D-DIGE) and protein identification

*L. biflexa* isolates were grown to a cell density of ~1 × 10^9^ cells/ml and harvested by centrifugation (10,000 × *g*, 10 min, 23°C). Cell pellets were rinsed in PBS and lysed in PBS supplemented with 1 X Complete Protease Inhibitor (Roche Applied Science) by 3 passes through a French pressure cell (16,000 lb/in^2^). Cell lysates were further fractionated into soluble and membrane-associated proteins by ultracentrifugation (100,000 × *g* 1 h, 4°C). The membrane-associated protein pellet was rinsed with PBS and suspended in PBS+PI with the aid of a glass tissue homogenizer (Kontes Glass Co.,Vineland, NJ). Protein concentrations were determined by a modified Lowry protein assay with bovine serum albumin as a standard.

For DIGE analysis of membrane-associated proteins, 50 ug of *L. biflexa* wild-type or the Δ*batABD* isolate was labeled with either 400 pmol Cy3 or Cy5 (CyDye minimal dye labeling kit, GE Healthcare) for 30 min on ice. As an internal control, a mixture of 25ug of the wild-type and 25 ug of the Δ*batABD* samples were labeled with Cy2 for 30 min on ice. All labeling reactions were performed in DIGE labeling solutions consisting of 7 M Urea, 2M Thiourea, and 4% CHAPS in 10 mM Tris (pH 8.5). The labeling reaction was quenched by adding 1 ul of 10 mM lysine and incubating for 10 min on ice. To ensure that observed differences were not due to artifacts from preferential dye binding to proteins, several coupled samples were labeled by dye switching. Labeled proteins were stored at −20°C in the dark until isoelectric focusing.

Cy-dye labeled samples for comparison were mixed and DTT and IPGphore 3–10 buffer were added at final concentrations of 100 mM and 1.0%, respectively. The volume of each set was brought to 350 ul with isoelectric focusing solution C_4_TT [49] and applied to 18 cm 3–10 non-linear IPG strips (GE Healthcare). Strips were focused using the following parameters: 12 hr rehydration, 500 V for 1 hr, 1000 V for 1 hr, 1500 V for 1 hr, 4000 V for 1 hr, and 8000V for 60,000 Vhr. Once focusing was complete, strips were stored at −80°C until equilibrated and separated in the second dimension by standard SDS-PAGE using 8-16% gradient gels (Jule, Inc., Milford, CT) [49].

After separation of DIGE-labeled strips by SDS-PAGE, gels were scanned in the glass plates using a three laser Typhoon 9400 variable mode imager (GE Healthcare, Piscataway, NJ) at 200 microns. Differences in protein spots were quantified using DeCyder 2-D Differential Analysis Software v7.2. Protein spots of interest were excised and processed for mass spectrometry as previously described [49]. Dried peptides were sent to the Protein Chemistry section of the NIAID Research Technologies Branch, NIH for identification as described below.

The recovered peptides were re-suspended in 5 ul of Solvent A (0.1% formic acid, 2% acetonitrile, and 97.9% water). Prior to mass spectrometry analysis, the re-suspended peptides were chromatographed directly on column, without trap clean-up. The bound peptides were separated at 500 nl/min generating 80–120 Bar pressure, using an AQ C18 reverse phase media (3 u particle size and 200 u pore) packed in a pulled tip, nano-chromatography column (0.100 mm ID × 150 mm L) from Precision Capillary Columns, San Clemente, CA. The chromatography was performed in-line with an LTQ-Velos Orbitrap mass spectrometer (ThermoFisher Scientific, West Palm Beach, FL) and the mobile phase consisted of a linear gradient prepared from solvent A and solvent B (0.1% formic acid, 2% water, and 97.9% acetonitrile) at room temperature. Nano LC-MS (LC-MS/MS) was performed with a ProXeon Easy-nLC II multi-dimensional liquid chromatograph and temperature controlled Ion Max Nanospray source (ThermoFisher Scientific) in-line with the LTQ-Velos Orbitrap mass spectrometer.

Mass calibration was performed as needed with the positive ion Cal Mix prepared as described by Thermo-Scientific and monitored by routine analysis of a 10 femtomole stock sample of BSA digest. Typical acceptable results for this analysis would yield a 2800 – 3300 Mascot score, 75 – 85% coverage and 0 - +/−4 ppm error when submitted to the Mascot server using Proteome Discoverer 1.3 using the Swiss Prot-Trembl data base.

Computer controlled data dependent automated switching to MS/MS by Xcalibur 2.1 software was used for data acquisition and provided the peptide sequence information. Data processing and databank searching were performed with PD 1.3 and Mascot software (Matrix Science, Beachwood, OH).

## Competing interests

The authors declare that they have no competing interests.

## Authors’ contributions

PES, MP, and PAR conceived of the study. PES carried out the molecular genetic studies, growth curve analyses, and drafted the manuscript. JAC carried out the proteomic experiments. DWD performed the microscopy. HHS and AS participated in the molecular genetic studies. MP participated in the design of the study and the molecular genetic studies. PAR participated in the manuscript and experimental design and helped to draft the manuscript. All authors read, edited and approved the final manuscript.

## Supplementary Material

Additional file 1 **Distribution of *bat *genes in the Spirochaetes.** Arrangement of *bat* genes in representative members of the Spirochaetes are shown compared to that found in *B. fragilis*. Gene fusions are denoted by *, and *batE* of *T. denticola* is significantly longer than in any other species examined (+), but does not appear to be a fusion with *batD*. (PDF 82 kb) Click here for file
