# Supplementary figures and images for: Characterization of the Bat proteins in the oxidative stress response of Leptospira biflexa
Source: BMC Microbiol. 2012 Dec 13;12:290. doi: 10.1186/1471-2180-12-290 (PMC3557215; doi:10.1186/1471-2180-12-290)

Supplementary Figure 1

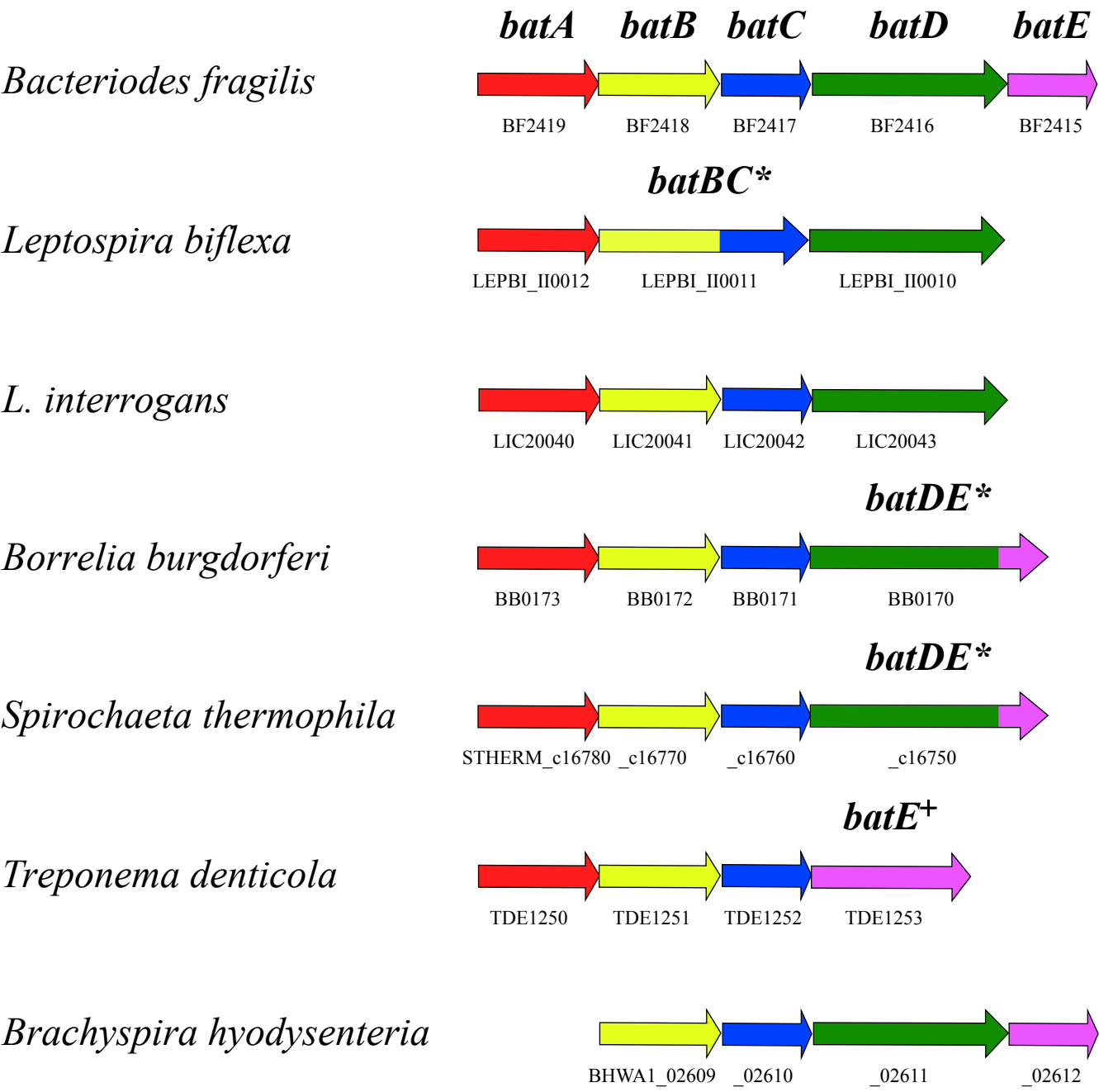

Supplement: Additional file 1 — Distribution of bat genes in the Spirochaetes. Arrangement of bat genes in representative members of the Spirochaetes are shown compared to that found in B. fragilis. Gene fusions are denoted by *, and batE of T. denticola is significantly longer than in any other species examined (+), but does not appear to be a fusion with batD. (PDF 82 kb) [file 1471-2180-12-290-S1.pdf]
